# Effect of antibiotics on bacterial populations: a multi-hierachical selection process

**DOI:** 10.12688/f1000research.9685.1

**Published:** 2017-01-17

**Authors:** José Luis Martínez

**Affiliations:** 1 Centro Nacional de Biotecnología, Consejo Superior de Investigaciones Científicas, Calle Darwin, Madrid, Spain

**Keywords:** antibiotic resistance, antibiotic, antibiotic-resistant mutants, antibiotic stress, clostridium difficile, mobile genetic element, microbiome, subinhibitory concentrations of antibiotics, biofilm

## Abstract

Antibiotics have been widely used for a number of decades for human therapy and farming production. Since a high percentage of antibiotics are discharged from the human or animal body without degradation, this means that different habitats, from the human body to river water or soils, are polluted with antibiotics. In this situation, it is expected that the variable concentration of this type of microbial inhibitor present in different ecosystems may affect the structure and the productivity of the microbiota colonizing such habitats. This effect can occur at different levels, including changes in the overall structure of the population, selection of resistant organisms, or alterations in bacterial physiology. In this review, I discuss the available information on how the presence of antibiotics may alter the microbiota and the consequences of such alterations for human health and for the activity of microbiota from different habitats.

## Introduction

Antibiotics are among the most successful drugs used in human therapy. In addition, they have been used for several decades in animal growth promotion, prophylaxis, metaphylaxis, treatment, and general farming production ^1– 4^. This wide antibiotic use has led to different habitats becoming polluted by a large range of concentrations of antibiotics ^5^. Since antibiotics are inhibitors of bacterial growth, this situation has an impact on the structure and the activity of bacterial populations. The effect of antibiotics on bacterial populations has mainly focused on the aspects related to human health, in particular the selection of antibiotic-resistant mutants and the acquisition, selection, and spread of antibiotic resistance genes ^6– 8^. While this has obvious relevance to the treatment of infectious diseases, other aspects of the roles that antibiotics may play in bacterial populations are much less studied in comparison ^9, 10^. In the field of human health, some studies have addressed the impact of antibiotic treatment on the global structure of the human gut microbiome ^11– 18^. These articles focus particularly on the general description of changes at the population level as well as on the selection of resistance genes. However, with recent work considering the gut microbiome itself as an organ, and taking into consideration that microbial composition may impact human physiology at different levels, more information on the consequences that changes to the human microbiome in the presence of antibiotics have on human health is still needed ^19– 22^. One aspect to be taken into consideration is that, when antibiotic treatment is needed, the effects of the antimicrobial on the microbiome should be considered as unavoidable side effects. Nevertheless, some work indicates that these effects can be mitigated by using compounds able to adsorb antibiotics in the gut ^23^. By using these compounds together with antibiotics, the concentration of the drug at the point of infection (unless in gut infections) will not change; however, it will be much lower at the gut and the microbiome should not be strongly altered.

One aspect that has received more attention in the last few years is the effect of antibiotics in environmental microbiota ^5, 9, 24^. Also, in this case, most studies focus on the aspects of this topic closer to human health, in particular how natural ecosystems, polluted or not with antibiotics, may be involved in the acquisition, selection, and spread of antibiotic resistance among human pathogens ^9, 24– 30^. This “one-health” approach is, of course, needed if we wish to fully understand the spread and maintenance of clinically relevant antibiotic-resistant microorganisms ^31^. Nevertheless, it is worth mentioning that fewer studies focus on the overall effect of antibiotics on the structure and productivity of environmental, not pathogenic, bacteria. Taking into consideration that all basic nutrient cycles in nature (carbon, nitrogen, oxygen, etc.) are based on the metabolism of microorganisms, learning whether or not antibiotic pollution may alter the right functioning of these cycles is of relevance. However, only some studies have addressed this relevant topic ^32– 35^. It is true that the concentrations of antibiotics are low in most ecosystems, but even low concentrations of antibiotics may trigger specific bacterial responses ^36– 39^, and analyzing such responses is a topic of interest.

In this review, I will discuss the multiple levels at which the presence of antibiotics may alter the structure of bacterial populations. Although the focus of the review will be the impact of such changes on human health, other more general aspects of the topic will be discussed as well.

## Antibiotics, natural compounds, and pollutants

Humankind has been using antimicrobial compounds for treating infections even before the discovery of microorganisms. However, these compounds did not belong to the type of chemical entities that are now known as antibiotics. Compounds such as mercury, lead, silver, or arsenic derivatives have been widely used. Even when the search for antimicrobials focused directly on inhibitors of microorganisms, the first industrially produced antibiotic was an organic derivative of arsenic, salvarsan. The first natural antibiotic was penicillin. However, the idea that soil (and water) can be a source of antimicrobials came from an ecological reflection: if soils are constantly polluted by pathogenic microorganisms, but soils are not a source of epidemics, there must be something in soils capable of killing human bacterial pathogens. This approach, proposed by Waskman and Woodruff ^40^, led to the identification of most of the antibiotic families currently in use in clinical practice. Indeed, although several natural antibiotics are chemically modified to improve their efficacy, few families, such as quinolones, have a synthetic origin, and even in this case, natural quinolones, some of them involved in cell-to-cell communication ^41^, have been found. Differing to xenobiotic compounds, which were not previously present in nature and can be refractory for their biodegradation, natural antibiotics are degradable. In addition, some microorganisms can subsist using antibiotics as a food resource ^42^. Since antibiotics are natural compounds, pollution by these drugs and the effect they have on bacterial populations are concentration-dependent problems. It is worth mentioning that we include under the name of antibiotics just those compounds that are useful for treating infections – in other words, those bacterial inhibitors without problems of toxicity and with pharmacokinetic/pharmacodynamic properties that allow their use in clinics. This does not necessarily mean that antibiotics are always inhibitors of microbial competitors at the low concentrations that they are naturally produced ^38^. Conversely, different microbial-produced compounds without the pharmacological properties required for treating an infection may serve in nature to inhibit the growth of competitors. Under this circumstance, it has been proposed that the effect of antibiotics on bacterial populations can be hormetic ^43– 45^ in character, beneficial at low concentrations and deleterious at the high ones usually present inside patients during treatment ^38, 46^. Distinguishing between these two situations is then critical for understanding the effect of antibiotics on bacterial populations.

## Multi-hierarchical antibiotic selection of bacterial populations

Since antibiotics are naturally produced compounds, it is expected that environmental microbial populations have adapted along their evolution to the presence of the natural concentrations of these antimicrobials ^47^. However, the constant discharge of antibiotics in nature may alter this homeostasis. Particularly important are the allocations in which antibiotic concentrations are higher: treated patients and animals. Other habitats in which high-level concentrations of antibiotics (and of resistance genes) can be found are waste-water treatment plants or rivers receiving domestic, hospital, farm, and industrial waters, soil at farms, water and sludge at fish farms, and manure ^5, 48^. In all cases, antibiotics can affect the structure of bacterial populations at different levels. First will be the population composition itself. Any bacterial species has a characteristic level of susceptibility to any given antimicrobial, which has been dubbed “intrinsic resistance” ^49– 51^. This means that, for any given concentration of antibiotic, a part of the population present in the microbiota (the most susceptible one) will be inhibited and another part will consequently increase their abundance. It is expected that a strong stressor (such as the presence of an antibiotic) will reduce diversity ^28, 52^, and this is likely to be true when the concentrations of the inhibitor are high. However, mild concentrations of antibiotics may produce an apparent increase in biodiversity, or at least the emergence of new taxons whose presence was minor before antibiotic stress ^53^, if the most predominant species present in the microbiome are susceptible and hence inhibited by such concentrations. The most detailed studies of the effect of antibiotics on the global composition of the microbiota have been performed studying the gut microbiota of humans and of experimental mammal models as well ^11, 12, 16, 18^. In all cases, a misbalance in the composition is observed upon treatment. Once treatment ends, a recovery of the composition of the microbiome is observed after some time. However, recent information indicates that, although the structure of the taxonomic groups is similar three months after treatment to the one observed before antibiotic application ^13^, the specific clones that re-colonize the gut are not the same as those before treatment. This means that while the overall structure of the population remains, the overall genomic content largely varies ^13^. These results indicate that the effect of antibiotics on the structure of the populations will remain long after they disappear from the polluted habitat. The disruption of the system as a consequence of antibiotic use can be followed by its reconstruction by an eco-equivalent microbiome. However, it does not mean that the functionality of the new clones is the same as the previous ones, a feature that may be of relevance for not only human health but also the productivity and biodegradative potential of environmental microbial populations.

At very high concentrations of antibiotics, the system may collapse and can be open for colonization by antibiotic-resistant microorganisms that otherwise would not be present in this ecosystem. This is the situation with *Clostridium difficile*, a major cause of gut infection in individuals following antibiotic treatment ^54, 55^ and whose infection is associated with a reduction in gut microbiota diversity ^56^. Given *C. difficile*’s low susceptibility to antibiotics, the best way for fighting recurrent infections caused by this pathogen is restoring the functionality of the gut microbiota via fecal transplantation ^57– 60^.

In addition to altering the overall structure of bacterial populations, the best-studied effect of antibiotics is the selection of antibiotic-resistant microorganisms. As stated above, selecting for intrinsic resistance will modify the taxonomic structure of a given ecosystem. Differing to that situation, the selection of mutants or bacteria carrying resistance genes acquired through horizontal gene transfer (HGT) will enrich some specific lineages that have acquired resistance. Here it is important to distinguish between mutation-driven and HGT-acquired resistance. The first is just vertically inherited and hence allows clonal expansion, whereas the second can be transferred both vertically and horizontally and hence can spread among the global population. In the case of HGT-driven resistance, different levels of selection can be foreseen. The gene is selected by the antibiotic, which produces the selection of the mobile genetic element (MGE) carrying it, the clone carrying the plasmid, and eventually the gene-exchange community to which this clone belongs if the resistance element spreads among its members ^61– 63^. As the consequence of this second-order selection, antibiotics may increase the success of some species and even of some specific clones in the community, somehow altering the overall physiology of the microbiome through the selection of a set of clones and genes. In this regard, it is worth mentioning that the number of genes present in nature and capable of conferring resistance upon their transfer to a heterologous host is several orders of magnitude larger than those currently found in human pathogens ^64, 65^. The human use of antibiotics has produced an explosive enrichment of a few so-called resistance genes present in MGEs, and now these are widespread all around the world ^66^.

## Short-term and long-term effects of antibiotics in bacterial populations

As stated above, antibiotics can alter the population structure of the microbiome (immediate effect), and while the overall structure of such microbiomes is recovered after some (usually a long) time, the genomic structure is not fully equivalent. As in the case of other strong stressors, this could be predicted; once an organism has been displaced, the same one will rarely re-colonize the habitat. This is not particularly relevant in the case of multicellular organisms. If a fire destroys a pine forest, obviously different pines will re-colonize the soil, but this does not have an impact on the overall activity of the system. However, in the case of microorganisms, the situation is dramatically different. Bacteria present a core genome that is shared by all members of the species and an accessory genome that is specific to each member of the species. The first encodes the most basic processes of the organism, and the second encodes the most adaptive ones: for instance, those that make the commensal bacteria *Escherichia coli* become a dangerous pathogen, those dealing with antibiotic resistance, or several involved in the biodegradation of toxic compounds. In this regard, although the basic activities encoded in the core genome will be restored, other activities can be lost when one clone is replaced by another. As stated, this situation is relevant for human health but can also be of relevance in other habitats such as waste-water treatment plants, where degradative bacteria can be important ^33, 53^. Current metagenomic techniques allow a broad taxonomic analysis of the populations as well as of the presence of specific genes in the microbiome. However, although some strategies have been implemented ^67– 73^, studies on genome reconstruction as well as gene-taxon binning (mainly in the case of mobile elements) are not easy to perform using currently available tools, at least in complex microbiota, which are the most frequently found. Under these circumstances, full information on the long-term effect of antibiotics in microbiome composition at the clonal level is still lacking.

Another (and better-studied) effect of antibiotics is the selection of antibiotic-resistant microorganisms. In this case, antibiotic pollution selects a set of mutants or genes (antibiotic resistance genes) that can be considered as pollutants themselves ^66, 74^ because they were not present (at least at the level they are now) in nature. The main difference between classical pollutants and resistant bacteria (or any type of microorganism at large) is that the first disappear over time and across space, whereas microorganisms and resistance genes are auto-replicative pollutants that can travel across long distances and remain over time ^75^. It has been proposed that the acquisition of antibiotic resistance confers a fitness cost that is reflected in a lower competitivity of the resistant microorganisms as compared with the susceptible one ^76, 77^. While this is true on occasion, it has been shown that antibiotic resistance might not reduce fitness but can even increase bacterial competitivity ^77, 78^. On top of that, resistant microorganisms can acquire compensatory mutations or physiological changes that restore their fitness ^79– 81^. Upon these conditions, it is not rare that bacteria carrying resistance genes are found in nearly any tested habitat, including domestic and wild animals, natural ecosystems, or untreated human volunteers, such as isolated Yanomami Amerindians, among others ^82– 92^. Human travel, interchanging of goods, climate alterations such as El Niño, and migratory birds, among other vectors, allow the intercontinental distribution of the auto-replicative pollutants that are antibiotic resistance and antibiotic resistance genes ^93– 97^.

While the acquisition of resistance may have the same ecological consequences for a human pathogen or for a non-pathogenic environmental microorganism, the consequences for human health are very different. Mutation-driven resistance is not a health risk if the resistant microorganism is not pathogenic. However, the acquisition of a resistance gene by an MGE is a risk for human health even when the MGE is present in an environmental microorganism ^64, 65^. It is important to remark that resistance genes currently present in MGEs were not present in human pathogens before the industrial production of antibiotics ^98^; they have originated from environmental microorganisms ^99– 101^. The farm-animal–to–human transfer of resistance has been discussed in detail, and farm animals are considered to be a reservoir of antibiotic resistance ^2^. Since the use of antibiotics for fish-farming challenges the fish, the water, and the sediment microbiota, this kind of multi-habitat selection situation might have had a relevant role in the first event of resistance acquisition by bacterial pathogens ^3^. In favor of this possibility is the finding that *Shewanella*, a waterborne organism, is likely the origin of antibiotic resistance determinants such as QnrA ^102^ or carbapenem-hydrolyzing oxacillinases ^103^, which are now widespread among human pathogens.

## Effect of subinhibitory concentrations of antibiotics on bacterial populations

Most studies on the effect of antibiotics on bacterial populations focus on inhibitory concentrations of the drugs. However, most populations confronted with antibiotics are challenged by subinhibitory concentrations of them. The study of the effect of such concentrations has shown that they can have deep effects on bacterial physiology. Indeed, in addition to triggering the expression of shock-response systems ^104– 107^, the antibiotics can induce specific bacterial responses. Some of them, dealing with the expression of virulence factors or motility, are specific to each family of drugs ^36^; however, some others seem to be more general. One of them is biofilm formation, which has been described to be triggered by different antibiotics ^36, 108– 111^. Since biofilms are more resistant to the action of antibiotics, it seems that this can be a protective response. In addition, it is important to remark that these physiological alterations may improve the bacterial colonization of surfaces. This improvement might have consequences for human health in the case of surface-associated infections (catheters, prosthesis, bladder, lung, etc.) and could also be relevant in natural ecosystems and in industries in which clogged pipelines can be problematic. All of these effects are transient and will disappear soon after removal of the antibiotic. However, even transient effects might produce an inheritable wave. It has been shown that antibiotics can increase mutation, recombination, gene transfer, and prophage induction, all of which have inheritable consequences ^112– 121^. Of course, to be evolutionarily relevant, these changes need to be fixed, and fixation is achieved only if bacteria are under selection. In this regard, although subinhibitory concentrations of antibiotics are not always considered to be direct drivers of evolution, it has been proposed that they can increase the evolvability of bacterial populations ^117, 122^.

This panorama has changed in the last few years. The classical view indicates that the selection of resistance can happen in a range of concentrations from the minimal inhibitory concentration, under which susceptible and resistant bacteria will grow, to the minimal preventive concentration, which inhibits the growth of resistant mutants. However, recent information indicates that subinhibitory concentrations of antibiotics can select antibiotic-resistant microorganisms ^123– 125^. While selection at high concentrations of antibiotics is based on the inhibition of the susceptible cells and hence a resistant population can be selected after few duplication events, both susceptible and resistant microorganisms grow at subinhibitory concentrations and selection is based on the differential fitness they present in the presence of the antimicrobial. This means that the selection of resistance requires, in this case, several duplications to allow the displacement of the susceptible population by the resistant one, which is fitter in the presence of an antibiotic. While it is true that there are several situations in which bacteria are under subinhibitory concentration, such as in the human body after treatment, these concentrations tend to be transient and it is difficult for a resistant population to be selected unless a constant selection pressure is implemented. There are, however, some situations in which this type of selection can be foreseen. One is in waste-water from hospitals or from antibiotic-producing plants. Another is in animal production when antibiotics are used as growth promoters. Indeed, the study of the metagenomes of pigs treated with antibiotics for long periods of time has shown their guts present an increase in Proteobacteria as well as in abundance and diversity of resistance genes, even some of them conferring resistance to antibiotics not administered in the study ^126^. These results raise the possibility that non-therapeutic use of antibiotics can be a major element in the selection of antibiotic-resistant bacteria in animals, which will eventually be more important than their therapeutic use.

Work on the effect of antibiotics on the behavior of bacterial populations usually takes into consideration just the antibiotic itself. However, recent work has shown that the presence of other stressors may modulate such effects. Usually, a second stressor increases the chances of acquiring resistance, but on other occasions the stressor antagonizes the selective pressure of the antibiotic ^127^. In the case of human health, this is particularly relevant when resistance to one antibiotic enhances the susceptibility to another (collateral sensitivity) because the use of such antibiotics together or in combination should reduce the chances of antibiotic resistance acquisition by human pathogens ^128– 130^.
